# Barriers to and Facilitators of the Implementation of Digital Mental Health Interventions as Perceived by Primary Care Decision Makers: Content Analysis of Structured Open-Ended Survey Data

**DOI:** 10.2196/44688

**Published:** 2023-06-26

**Authors:** Anders Brantnell, Serdar Temiz, Enrico Baraldi, Joanne Woodford, Louise von Essen

**Affiliations:** 1 Division of Industrial Engineering and Management Department of Civil and Industrial Engineering Uppsala University Uppsala Sweden; 2 Healthcare Sciences and e-Health Department of Women’s and Children’s Health Uppsala University Uppsala Sweden; 3 Division of Industrial Engineering and Management, Department of Civil and Industrial Engineering Uppsala University Uppsala Sweden

**Keywords:** digital mental health, implementation, barriers, facilitators, internet-based cognitive behavioral therapy, survey, decision makers

## Abstract

**Background:**

Digital mental health represents a way to increase access to evidence-based psychological support. However, the implementation of digital mental health in routine health care practice is limited, with few studies focusing on implementation. Accordingly, there is a need to better understand the barriers to and facilitators of implementing digital mental health. Existing studies have mainly focused on the viewpoints of patients and health professionals. Currently, there are few studies about barriers and facilitators from the perspective of primary care decision makers, that is, the persons responsible for deciding whether a given digital mental health intervention should be implemented in a primary care organization.

**Objective:**

The objectives were to identify and describe barriers to and facilitators of the implementation of digital mental health as perceived by primary care decision makers, evaluate the relative importance of different barriers and facilitators, and compare barriers and facilitators reported by primary care decision makers who have versus have not implemented digital mental health interventions.

**Methods:**

A web-based self-report survey was conducted with primary care decision makers responsible for the implementation of digital mental health in primary care organizations in Sweden. Answers to 2 open-ended questions about barriers and facilitators were analyzed through summative and deductive content analysis.

**Results:**

The survey was completed by 284 primary care decision makers—59 (20.8%) decision makers representing implementers (ie, organizations that offered digital mental health interventions) and 225 (79.2%) respondents representing nonimplementers (ie, organizations that did not offer digital mental health interventions). Overall, 90% (53/59) of the implementers and 98.7% (222/225) of the nonimplementers identified barriers, and 97% (57/59) of the implementers and 93.3% (210/225) of the nonimplementers identified facilitators. Altogether, 29 barriers and 20 facilitators of implementation were identified related to guidelines; patients; health professionals; incentives and resources; capacity for organizational change; and social, political, and legal factors. The most prevalent barriers were related to incentives and resources, whereas the most prevalent facilitators were related to the capacity for organizational change.

**Conclusions:**

A number of barriers and facilitators were identified that could influence the implementation of digital mental health from the perspective of primary care decision makers. Implementers and nonimplementers identified many common barriers and facilitators, but they differ in terms of certain barriers and facilitators. Common and differing barriers and facilitators identified by implementers and nonimplementers may be important to address when planning for the implementation of digital mental health interventions. For instance, financial incentives and disincentives (eg, increased costs) are the most frequently mentioned barrier and facilitator, respectively, by nonimplementers, but not by implementers. One way to facilitate implementation could be to provide more information to nonimplementers about the actual costs related to the implementation of digital mental health.

## Introduction

### Background

Common mental health problems, such as depression and anxiety, represent substantial global health challenges [1]. Depression is estimated to be the third-leading cause of disability globally [2], and approximately 29% of all people will be affected by an anxiety disorder during their lifetime [3]. Cognitive behavioral therapy (CBT) delivered face to face is a common and effective treatment for depression and anxiety [4]. However, face-to-face treatments require large organizational resources and visits to health care providers’ offices. Digital mental health represents a way to improve access to care [5] and decrease care costs [6]. Digital mental health can be defined as mental health services and interventions delivered through the internet, telephone, or connected technologies [7]. Internet-administered CBT (ICBT) is a form of digital mental health and has been shown to be as effective as face-to-face CBT for the treatment of depression and anxiety [4]. However, although there is a growing body of research showing the efficacy [8] and cost-effectiveness [9] of ICBT for common mental disorders such as depression and anxiety [10-12], studies of the implementation of ICBT in routine health care practice are limited [13].

To enable implementation and increase access to digital mental health, there is a need to understand aspects that may influence the implementation of digital mental health interventions, that is, barriers to and facilitators of implementation. Studies of the implementation of digital mental health are relatively scarce [13], and only a few reviews have identified barriers to and facilitators of implementation [14-18]. By focusing only on the views of patients and health professionals, existing studies have identified barriers such as negative attitudes toward digital mental health [14-16], the lack of suitability of digital mental health for various mental health problems [14-16], low computer literacy [15-17], the lack of training for health professionals [17], and existing infrastructure [17]. Some identified facilitators include training for health professionals [14,16], mild symptoms [14,16], and ease of use [14]. A recent theoretical overview of digital mental health interventions [18] identified barriers such as privacy and security concerns, usability issues from patients’ point of view, patients’ knowledge and skills, and clinicians’ skills and capabilities. One qualitative study exploring mental health professionals’ perspectives about digital mental health implementation identified barriers, such as negative attitudes of clinicians, existing infrastructure, and “one solution does not fit all,” and facilitators, such as the packaging solutions [19]. Furthermore, continued implementation is also a challenge, with a recent review identifying 131 empirical studies of the rapid deployment of digital mental health interventions as a response to the COVID-19 pandemic, with several barriers identified regarding long-term sustainability [20].

Given the few studies focusing on the implementation of digital mental health, it is reasonable to look broadly into digital health implementation. Existing studies in the area have identified several factors that could hinder or facilitate the implementation of interventions. For example, a review studied the factors influencing the adoption of digital applications by health care professionals and identified 101 studies exploring barriers to and facilitators of implementation [21]. Some of the most frequent facilitators of implementation were the usefulness of the innovation and compatibility, whereas some of the most frequent barriers were related to the lack of knowledge among health care professionals and the lack of compatibility [21].

A review of studies (n=16) of the implementation of digital technologies to support patients with amyotrophic lateral sclerosis identified several facilitators of implementation, such as positive attitudes of health care professionals and the training of health care professionals, and barriers, such as negative attitudes of health care professionals and feasibility [22]. Another review of the barriers to the use of digital health by older adults identified 57 studies detailing barriers, with the most frequent barriers being the lack of interest and cost of use [23]. A review focusing on digital health for self-management of hypertension included studies (n=14) that identified barriers to and facilitators of implementation [24]. Some of the most frequent facilitators were access to technology, patient knowledge, and ease of use. In contrast, some of the most frequent barriers were the lack of evidence and added workload [24].

However, none of the digital mental health or digital health reviews identify barriers and facilitators experienced by health care decision makers, that is, the professionals who take the decision to implement or disregard new solutions. Although not included in reviews, there are some qualitative studies that have explored the barriers and facilitators experienced by health care decision makers. A recent qualitative study in Sweden explored policy makers’ views (ie, those who formulate rules and regulations regarding digital health at the regional level, such as politicians) about barriers to and facilitators of the implementation of digital health [25]. Some identified barriers included uncertainty about the impact of digital health on health professionals and the lack of resources for digital health, whereas facilitators included citizens’ preferences and a strong societal push for digital health [25]. Another qualitative study focusing on barriers to the implementation of digital mental health in the United Kingdom, as experienced by health decision makers (health commissioners), identified barriers such as the lack of decision-maker knowledge about the technology, digital literacy among users and decision makers, high risk of investing in digital mental health, funding issues, and digital interventions not being suitable for all patients [26]. In addition, our previous findings from a web-based cross-sectional quantitative survey about barriers to and facilitators of the implementation of ICBT experienced by primary care decision makers identified a number of barriers to and facilitators of implementation. However, the quantitative survey focused on comparing barriers and facilitators between implementers and nonimplementers but did not capture frequency and thus decision makers’ preferences [27].

### Objectives

The objectives of this study were (1) to identify and describe barriers to and facilitators of the implementation of digital mental health as perceived by primary care decision makers; (2) to evaluate the relative importance of the barriers and facilitators; and (3) to compare the barriers and facilitators between primary care decision makers who have implemented versus have not implemented digital mental health.

## Methods

### Study Design

A web-based self-report survey was conducted between February 2016 and May 2016 with decision makers responsible for the implementation of digital mental health in primary care organizations in Sweden. The survey focused on the implementation of ICBT for depression and anxiety disorders. Answers to the structured open-ended questions in the survey are reported in this paper. Results from the rest of the survey have been reported elsewhere [27].

### Setting

Sweden was one of the first countries to conduct research on ICBT for depression and anxiety [28]. Swedish national clinical guidelines recommend that CBT and ICBT be provided to adults with mild and moderate levels of depression and anxiety [29]. However, the implementation of ICBT is still in its infancy [30].

Sweden is divided into 21 geographically spread regions that are responsible for health care provision. Each region has several private and public primary care organizations that are publicly funded and thus operate under the same conditions, for instance, in terms of financial resources and adherence to national guidelines. Primary care is the first point of care for patients with mental health problems. The size of the primary care organizations varies in terms of listed patients ranging from 3000 to 30,000. In addition to publicly funded primary care organizations, there are private companies specialized in digital mental health.

Primary care organizations are able to access ICBT through three means: (1) contracting a private company to deliver digital mental health, including support; (2) procuring ICBT program licenses from companies and providing support by themselves; or (3) connecting to the Platform for Support and Care run by the Swedish Association of Local Authorities. Through the Platform for Support and Care, primary care organizations can access ICBT programs developed by private companies or other organizations. There is a cost for connecting to the Platform for Support and Care and for purchasing the treatment programs with or without therapist support. There is also a cost to patients. In the Stockholm Region (one of the 21 regions in Sweden), a web-based meeting with therapist support costs approximately €25 (US $26.7), and costs >€130 (US $138.8) for a patient during the same year will be covered by the public insurance. Furthermore, for patients, it is possible to access ICBT through a private company; for instance, a company charges €75 (US $80.1) for the first meeting and, subsequently, €75 (US $80.1) per week.

As a response to the COVID-19 pandemic, there has been a rapid increase of digital health solutions globally [20]. Available data from 10 regions (including many of the large regions in Sweden) show that 1781 treatments for digital mental health started in 2019 and 4573 started in 2022, when the pandemic had passed, and indicate a modest increase in the provision of digital mental health treatments since the COVID-19 pandemic (for details, refer to Multimedia Appendix 1 [30-32]). Available data do not cover all regions, and according to estimations, 17,800 digital mental health treatments started in 2021 [31]. However, when compared with the number of registered cases of major depression (670,980/5,397,675, 12.43%) and anxiety disorders (536,279/5,397,675, 9.94%) in the first Primary Care Registry in Sweden [33] (common mental health conditions that may be treated with ICBT), the number of digital mental health treatments started still appears to be very low.

### Recruitment and Study Procedures

Study participants were directors of Swedish primary care organizations. A list of 1156 primary care organizations was compiled, and an invitation was sent to all decision makers. Invitations were initially sent through regular mail and were followed up via telephone and emails. Invitations included a letter explaining the survey and information needed to participate, link to the survey, participation number, and password. Participants who completed the survey provided informed consent through the survey platform (SurveyMonkey [Momentive Global Inc]). No incentives were offered for survey completion. Participants who did not complete the survey within 2 weeks received up to 2 telephone reminders and 1 email. Details about the study procedures are reported elsewhere [27].

### The Survey

Answers to the 2 open-ended questions in the survey are reported in this study. The following questions were posed:

1. According to your understanding, what are the most important factors that hinder the introduction of ICBT programs? Please indicate a maximum of 5 factors.
2. According to your understanding, what are the most important factors that facilitate introduction of ICBT programs? Please indicate a maximum of 5 factors.

Questions were posed at the end of the survey and were preceded by 37 Likert-scale questions about barriers and facilitators (for details about the survey, refer to the paper by Brantnell et al [27]).

### Data Analysis

Data analysis follows summative content analysis, which is a suitable approach to analyze large amounts of open-ended survey data [34]. As there is an abundance of studies of barriers to and facilitators of the implementation of health care interventions, deductive content analysis complemented the summative content analysis [35] guided by the comprehensive integrated checklist of determinants of practice (the Tailored Implementation in Chronic Diseases [TICD] checklist) [36]. The TICD checklist [36] divides barriers and facilitators into each of the seven domains: (1) guidelines; (2) health professionals; (3) patients; (4) professional interaction; (5) incentives and resources; (6) capacity for organizational change; and (7) social, political, and legal factors. The checklist is based on a rigorous review of existing studies of barriers to and facilitators of implementation [36] and provides a good basis for identifying barriers to and facilitators of implementation. The survey that was reported by Brantnell et al [27] adjusted the TICD checklist according to the Swedish conditions and the Likert-scale approach of the survey questions and thus originated from 5 domains. With open-ended structured data, there was no need to adjust the original TICD checklist because the domains, barriers, and facilitators that would be irrelevant would not be included in the analysis. The analysis was conducted in 6 steps.

First, following a summative content analysis approach [34], data were divided into four small blocks administered through separate Microsoft Excel files: (1) barriers mentioned by implementers (ie, decision makers of organizations that had implemented ICBT); (2) barriers mentioned by nonimplementers (ie, decision makers of organizations that had not implemented ICBT); (3) facilitators mentioned by implementers; and (4) facilitators mentioned by nonimplementers. Second, the Leximancer software was used to identify the most frequent words in each of the 4 data blocks. Subsequently, Excel files were searched for each of the frequent words. To identify possible synonyms for each word, a web-based database, Synonymer.se [37] was used, and the words were added in the search. When applicable, some area-specific synonyms that were not identified by Synonymer.se [37] were added. For example, synonyms to “staff” were “therapist,” “speech therapist,” “psychologist,” “the one treating patients” (*behandlare* in Swedish), and “medical doctor.”

Third, all hits in the Excel files were marked, and frequent words and phrases were copy-pasted into a Microsoft Word file. The frequency of the copy-pasted words and phrases was recorded. At this stage, no interpretation of data was conducted, but similar words and phrases were combined into large units. Fourth, the words and phrases were translated into English to try to maintain the Swedish phrasing while also capturing the essence of the words and phrases. Fifth, following deductive thematic analysis [35], two researchers independently placed the words and phrases into the TICD checklist under suitable domains (eg, guidelines) and barriers and facilitators (eg, the accessibility of the intervention). Sometimes, the respondents did not provide the direction of the barrier and facilitator. An example of this could be “health professional interest.” In such cases, we modified the word or phrase to include the direction such as “health professionals not interested” (if provided as an answer regarding barriers) or “health professionals interested” (if provided as an answer regarding facilitators). There are some overlaps in the TICD checklist, and thus, the 2 researchers placed some words and phrases under >1 barrier and facilitator. If words and phrases did not fit with existing barriers and facilitators, new barriers and facilitators were created and integrated into the checklist. To increase the credibility of the deductive analysis, coding was conducted by 2 independent coders, which is a recommended procedure when using an existing checklist or framework [38].

An internal workshop was conducted to compare and discuss the outcomes from the deductive analysis. All disagreements were solved through discussion during the workshop. A decision was made to place all words and phrases that lacked a subject (ie, the actor experiencing the barrier or facilitator) such as “leadership” under leadership barriers and facilitators because it was the decision makers who answered the survey. In many cases, respondents provided the subject such as patients or health professionals, and thus, when the subject was missing, a reasonable conclusion was that the words and phrases referred to leadership. While placing words and phrases into the TICD checklist, their frequencies were recorded. All words and phrases mentioned by at least 2 participants were included. Throughout the process, all the authors were involved in discussing and following up on the analysis to increase rigor and trustworthiness [39]. Finally, following the deductive content analysis, the frequencies of each barrier and facilitator were summarized using descriptive statistics, and a comparison between implementers and nonimplementers was conducted. The number of barriers and facilitators was counted for implementers and nonimplementers by adding all words and phrases relating to specific barriers and facilitators.

### Ethics Approval

The study was performed in accordance with the Swedish ethical law and the Declaration of Helsinki. The ethical review board of Sweden, Uppsala, approved the study (application number 2015/461).

## Results

### Respondents

A total of 1156 survey invitations were sent, of which 1130 (97.8%) were shown to be eligible. Noneligible answers that were excluded were duplicate answers (13/26, 50%), bankruptcy or closed down (10/26, 38%), and not a primary care organization (3/26, 12%). A total of 284 decision makers answered the 2 open-ended survey questions.

### Characteristics of the Decision Makers

Most decision makers (277/284, 97.5%) were health care center directors or chief executive officers. The 3 most frequent professions of decision makers were nurse (154/284, 54.2%), general practitioner (59/284, 20.8%), and physiotherapist (20/284, 7%). Among the respondent organizations, 20.8% (59/284) provided ICBT and were thus implementers. Overall, 63% (179/284) of the decision makers represented public organizations and the remaining represented private organizations. Both types of organizations were publicly funded.

### Barriers to and Facilitators of Implementation of ICBT

#### Overview

Altogether, 59 implementers responded, of which 57 (97%) listed the facilitators of implementation and 53 (90%) listed the barriers to implementation. In contrast, 225 nonimplementers responded, of which 210 (93.3%) listed the facilitators of implementation, whereas 222 (98.7%) listed the barriers to implementation.

In total, 29 barriers to and 20 facilitators of the implementation of ICBT were identified (Tables 1 and 2), and these were grouped within 6 domains based on the TICD checklist (guidelines; health professionals; patients; incentives and resources; capacity for organization change; and social, political, and legal factors). No barriers and facilitators were mentioned regarding the seventh domain in the TICD checklist, namely, professional interaction. For detailed outcomes of the summative and deductive content analysis, refer to Multimedia Appendix 2. All the barriers and facilitators are presented in Tables 1 and 2. The most frequently mentioned barriers were related to incentives and resources (ie, availability of necessary resources; 14/53, 26%) and capacity for organizational change (ie, capable leadership; 14/53, 26%; Table 1), whereas the most frequently mentioned facilitators were related to capacity for organizational change (ie, assistance for organizational change; 57/210, 27.1%; Table 2).

**Table 1:** Barriers mentioned by implementers and nonimplementers, distributed according to the Tailored Implementation in Chronic Diseases checklist.

Domains and barriers		Implementers (n=53), n (%)	Nonimplementers (n=222), n (%)
**Guidelines**			
	Compatibility	8 (15.1)	—^a^
	Feasibility	—	23 (10.4)
	Strength of the recommendation	—	20 (9)
	Accessibility of the intervention	2 (3.8)	4 (1.8)
	Quality of evidence supporting the recommendation	2 (3.8)	2 (0.9)
	Effort	—	4 (1.8)
	Clarity	—	2 (0.9)
	Cultural appropriateness	—	2 (0.9)
	Trialability	—	2 (0.9)
**Health professionals**			
	Intention and motivation	10 (18.9)	23 (10.4)
	Nature of the behavior	6 (11.3)	2 (0.9)
	Attitudes	—	14 (6.3)
	Skills needed to adhere	2 (3.8)	2 (0.9)
	Awareness and familiarity with the recommendation	—	5 (2.2)
**Patients**			
	Patient motivation and interest	4 (7.5)	15 (6.8)
	Patient behavior	3 (5.7)	5 (2.2)
	Patient preferences	2 (3.8)	11 (4.9)
	Patient beliefs and knowledge	—	4 (1.8)
**Incentive and resources**			
	Availability of necessary resources	14 (26.4)	29 (13.1)
	Financial incentives and disincentives	5 (9.4)	33 (14.9)
	Information system	3 (5.7)	15 (6.8)
	Availability of supporting infrastructure	3 (5.7)	11 (4.9)
**Capacity for organizational change**			
	Capable leadership	14 (26.4)	53 (23.9)
	Organizational readiness	6 (11.3)	25 (11.3)
	Assistance for organizational change	5 (9.4)	11 (4.9)
	Mandate, authority, and accountability	2 (3.8)	—
	Regulations, rules, and policies	—	2 (0.9)
**Social, political, and legal factors**			
	Health care system	—	18 (8.1)
	Contracts	—	3 (1.4)

^a^Not available.

**Table 2:** Facilitators mentioned by implementers and nonimplementers, distributed according to the Tailored Implementation in Chronic Diseases checklist.

Domains and facilitators			Implementers (n=57), n (%)		Nonimplementers (n=210), n (%)
**Guidelines**					
	Accessibility of the intervention	8 (14)		36 (17.1)	
	Feasibility	5 (8.8)		14 (6.6)	
	Strength of the recommendation	4 (7)		2 (1)	
	Observability	3 (5.3)		—^a^	
	Clarity	3 (5.3)		—	
	Compatibility	—		3 (1.4)	
**Health professionals**					
	Intention and motivation	11 (19.3)		24 (11.4)	
**Patients**					
	Patient motivation and interest	4 (7)		5 (2.4)	
	Patient beliefs and knowledge	2 (3.5)		6 (2.9)	
	Patient behavior	—		6 (2.9)	
**Incentives and resources**					
	Financial incentives and disincentives	2 (3.5)		37 (17.6)	
	Availability of necessary resources	5 (8.8)		9 (4.3)	
	Information system (people, platform, and technology combined)	3 (5.3)		12 (5.7)	
	Availability of supporting infrastructure	2 (3.5)		2 (1)	
	Nonfinancial incentives and disincentives	2 (3.5)		2 (1)	
**Capacity for organizational change**					
	Assistance for organizational change	11 (19.3)		57 (27.1)	
	Capable leadership	5 (8.8)		44 (21)	
	Relative strength of supporters and opponents	2 (3.5)		7 (3.3)	
	Mandate, authority, and accountability	—		2 (1)	
**Social, political, and legal factors**					
	Health care system	—		2 (1)	

^a^Not available.

#### Guidelines

Overall, 9 barriers and 6 facilitators were identified regarding the guidelines for ICBT interventions. Implementers most often mentioned the lack of compatibility with existing technology (8/53, 15%) as a barrier. In contrast, nonimplementers most often mentioned the feasibility of the intervention (ie, the extent to which the intervention is practical; 23/222, 10.4%). Of the 9 barriers identified, 2 (the accessibility of the intervention and quality of evidence supporting the recommendation) were mentioned by both implementers and nonimplementers. Both implementers (8/57, 14%) and nonimplementers (36/210, 17.1%) most often mentioned the accessibility of the intervention as a facilitator. Of the 6 facilitators identified, 3 (the accessibility of the intervention, feasibility, and strength of recommendation) were mentioned by both implementers and nonimplementers.

#### Health Professionals

Overall, 5 barriers and 1 facilitator were identified related to health professionals. Implementers (10/53, 19%) and nonimplementers (23/222, 10.4%) most often mentioned the lack of intention and motivation as barriers. Of the 5 barriers identified, 3 (intention and motivation, the nature of the behavior, and skills needed to adhere) were mentioned by both implementers and nonimplementers. Both implementers and nonimplementers mentioned only 1 facilitator—intention and motivation (11/57, 19% for implementers and 24/210, 11.4% for nonimplementers).

#### Patients

Overall, 4 barriers and 3 facilitators were identified related to patients. Implementers (4/53, 8%) and nonimplementers (15/222, 6.8%) most often mentioned the barrier, lack of patient motivation and interest. Of the 4 barriers identified, 3 (patient motivation and interest, patient behavior, and patient preferences) were mentioned by both implementers and nonimplementers. The most frequently mentioned facilitator by implementers was patient motivation and interest (4/57, 7%). In contrast, the most frequently mentioned facilitator by nonimplementers were patient beliefs and knowledge (6/210, 2.9%) and patient behavior (6/210, 2.9%). Of the 3 facilitators identified, 2 (patient motivation and interest and patient beliefs and knowledge) were mentioned by both implementers and nonimplementers.

#### Incentives and Resources

Overall, 4 barriers and 5 facilitators were identified regarding incentives and resources. Implementers most often mentioned the barrier, the availability of necessary resources (14/53, 26%). In contrast, nonimplementers most often mentioned the barriers, financial incentives and disincentives (33/222, 14.9%) and the information system (33/222, 14.9%). All the 4 barriers identified were mentioned by both implementers and nonimplementers. Implementers most often mentioned the facilitator, the availability of necessary resources (5/57, 9%), whereas nonimplementers most often mentioned the facilitator, financial incentives and disincentives (37/210, 17.6%). All the 5 facilitators identified were mentioned by both implementers and nonimplementers.

#### Capacity for Organizational Change

Overall, 6 barriers and 5 facilitators were identified regarding capacity for organizational change. Both implementers and nonimplementers most often mentioned the barrier, capable leadership (ie, leadership interest and knowledge; 14/53, 26% for implementers and 53/222, 23.9% for nonimplementers). Of the 5 barriers identified, 3 (capable leadership, organizational readiness, and assistance for organizational change) were mentioned by both implementers and nonimplementers. Organizational readiness was not part of the TICD checklist but originated from the summative content analysis and was added to the checklist. Implementers (11/57, 19%) and nonimplementers (57/210, 27.1%) most often mentioned the facilitator, assistance for organizational change. Of the 4 facilitators identified, 3 (assistance for organizational change, capable leadership, and relative strength of supporters and opponents) were mentioned by both implementers and nonimplementers.

#### Social, Political, and Legal Factors

Overall, 2 barriers and 1 facilitator were mentioned by nonimplementers related to social, political, and legal factors. The barrier that was most often mentioned was the health care system (18/210, 8.6%). The health care system was not part of the TICD checklist but was added based on the summative content analysis. The only facilitator was the health care system (2/210, 0.9%).

## Discussion

### Principal Findings

A total of 284 decision makers participated in the survey and provided answers to 2 open-ended questions. The majority of respondents (277/284, 97.5%) were health care center directors or chief executive officers. The 3 most common professions among the decision makers were nurses (154/284, 54.2%), general practitioners (59/284, 20.8%), and physiotherapists (20/284, 7%). Out of all the organizations represented, 20.8% (59/284) offered ICBT and were labeled as implementers. Among the implementers, 90% (53/59) identified barriers to implementation, while 97% (57/59) listed facilitators of implementation. On the other hand, among the nonimplementers, 98.7% (222/225) listed barriers to implementation and 93.3% (210/225) listed facilitators of implementation. In total, 29 barriers to and 20 facilitators of implementing ICBT were identified.

Findings identified barriers to and facilitators of the implementation of digital mental health related to 6 domains in the TICD checklist: guidelines; health professionals; patients; incentives and resources; capacity for organizational change; and social, political, and legal factors. First, we conducted summative content analysis based on the responses. During this phase, we were able to capture barriers and facilitators, as expressed by the respondents. Second, we connected the responses with the TICD checklist [36]. No barriers or facilitators were identified related to the TICD checklist domain, professional interaction. In addition, we identified 3 new barriers and facilitators that were added to the TICD checklist: the availability of supporting infrastructure (domain: incentives and resources), organizational readiness (domain: capacity for organizational change), and the health care system (domain: social, political, and legal factors).

Findings show that the most frequently mentioned barriers related to availability of necessary resources (14/53, 26%) and capable leadership (14/53, 26%), whereas the most frequently mentioned facilitators related to assistance for organizational change (57/210, 27.1%). Existing studies of the implementation of digital mental health [26] and digital health [25] interventions focusing on decision makers imply that the availability of necessary resources is an important barrier to implementation. This barrier (availability of necessary resources) is further supported by existing studies of policy makers’ use of evidence [39] and barriers to implementation related to third-sector actors providing health care [40]. Our findings align with a review focusing on health professionals’ views that identified important barriers and facilitators related to organizations, systems, and health professionals including assistance for organizational change [16]. However, our findings also suggest that assistance for organizational change also relates to decision makers. Education and support, which are important components of organizational change (for details, refer to the codes in Multimedia Appendix 2—under the column heading *Words and phrases mentioned by respondents*, grouped as *Assistance for organizational change*), have been identified as barriers in existing studies focusing on implementation facilitators for third-sector actors providing health care [40] and studies of health policy makers’ use of evidence [41].

Assistance for organizational change and capable leadership are closely related, and thus, it is unsurprising that capable leadership is one of the most frequently mentioned barriers to implementation. That is, if managers are not trained and educated, they will not be able to support implementation. Increasing knowledge requires an implementation strategy [42], and in general, implementation strategies are reported to have between 4% and 10% effect sizes in changing behavior [43]. Furthermore, the lack of knowledge could be dependent on other factors such as attitudes and outcome expectations, and thus, addressing these barriers is also needed [42]. Therefore, it is not likely that COVID-19 and similar disruptions could wipe out complex and sizable barriers to implementation, and thus, a reasonable assumption is that many barriers are persistent and require structured implementation efforts rather than sudden external pressure. These assumptions are also supported in existing studies of barriers to sustain digital mental health interventions after the COVID-19 pandemic [20].

Comparing our findings with those of existing studies of frequent barriers and facilitators, we underscore 3 important findings. First, existing studies of digital mental health have identified very few barriers and facilitators related to guidelines or the therapy in itself, such as the strength of the recommendation (ie, 1 solution does not fit all) [19,26]. However, the frequently mentioned guideline-related barriers and facilitators in our findings, the lack of compatibility [21], feasibility [22,44], and the accessibility of the intervention [24], are well established in existing studies of digital health implementation. Second, existing studies of digital mental health [26] and digital health [25] with focus on decision makers do not raise the importance of health professionals’ intention and motivation as both barriers to and facilitators of implementation. In our findings, this barrier and facilitator was the most frequently mentioned related to health professionals, and it is also identified in existing studies of digital mental health [19] and digital health [22]. Third, patient’s motivation and interest are identified as important barriers to and facilitators of implementation in existing studies of digital health implementation [23,24], but they are not very prevalent among our findings regarding the implementation of digital mental health from the perspective of decision makers.

Implementers and nonimplementers identified a number of similar barriers and facilitators relating to 4 (health professionals, patients, incentives and resources, and capacity for organizational change) of the 6 domains. Most similarities were identified in relation to incentives and resources. However, there were differences in how frequently these barriers were mentioned. Implementers report availability of necessary resources as the most frequent barrier and facilitator, whereas these are not the most frequently reported by nonimplementers. These findings imply that the implementation of digital mental interventions is not dependent on available resources, albeit may be hindered by lack of them. This, in turn, could be encouraging for nonimplementers that lack the necessary resources to invest in digital mental health. One way to facilitate implementation could be to communicate to nonimplementers, especially persons with budgetary responsibilities, that maintaining implementation requires additional resources. Whether maintaining implementation requires extra resources is an empirical question for further studies.

Financial incentives and disincentives are the barriers and facilitators most frequently mentioned by nonimplementers but not by implementers, which implies that nonimplementers perceive structural hinders for implementation related to financial aspects such as the reimbursement system and increased costs. In contrast, implementers do not perceive the financial incentives and disincentives as highly problematic, which, in turn, could be motivating for nonimplementers that assume these to be sizable barriers to implementation. One way to facilitate implementation could be to provide more information to nonimplementers regarding the actual costs related to the implementation of digital mental health. Whether the benefits of digital mental health interventions are related to financial incentives or other aspects such as improved care warrants further studies.

The most obvious difference between implementers and nonimplementers was found in barriers and facilitators related to guidelines. The 2 barriers most frequently mentioned by nonimplementers related to guidelines are the feasibility and strength of recommendation, whereas these are not mentioned by implementers. Whether these 2 barriers are real barriers based on experience or only based on assumptions is unclear; however, overcoming these barriers, for instance, through education could improve the possibilities for implementation, and thus, it could be beneficial to educate nonimplementers regarding the feasibility and strength of the recommendation. Implementers most frequently mention compatibility as a barrier related to guidelines, whereas nonimplementers do not mention this barrier, which implies that implementers perceive that there is not an optimal fit between the digital mental health intervention and existing work practices. This type of barrier is difficult to overcome because it is at the core of the intervention, that is, starting to use the intervention requires work with computers and thus requires a more complex implementation strategy targeting possible barriers such as digital literacy, attitudes toward digital mental health, and adaptation of existing work routines to accommodate the provision of digital mental health.

### Limitations

Our study has some limitations. First, we collected structured, open-ended data using a survey, which is not an optimal way to gain an in-depth understanding of the barriers and facilitators because no follow-up questions can be posed. However, we followed a well-structured and rigorous analysis process that should be able to provide a good overview of barriers to and facilitators of ICBT implementation in Sweden from the perspective of decision makers. Second, we collected data from 1 country. Sweden has publicly funded health care, with good access to care. There could be certain contextual differences between different health care systems, but some barriers and facilitators such as capable leadership could apply to several contexts and digital mental health more generally. Whether capable leadership and the other identified barriers and facilitators also apply to other health care systems and technologies is an empirical question for further studies.

Third, our data were collected in 2016. However, these data are still relevant for several reasons: neither the intervention nor the context has changed substantially since 2016. Swedish primary care organizations can still access ICBT in several ways, and it is often the primary care director who can make the decision regarding whether to offer ICBT. We acknowledge that during the past years, the technology has probably matured and could have become less costly and more easily available. However, it is unlikely that disruptions such as COVID-19 and technology advancements have overcome the complex and sizable barriers to implementation that were identified, and thus, a reasonable assumption is that many barriers are persistent and require structured implementation efforts. For instance, it is unlikely that there has been substantial increase in available resources for digital mental health, and although regions in Sweden are investing in digital innovations, mental health has not been their priority [45]. Similarly, leadership and public health workforce capacity building requires structured and complex implementation efforts [46].

Moreover, despite expectations of a massive increase of digital mental health treatments after the COVID-19 pandemic, the number of treatments in Sweden was still modest during the COVID-19 pandemic (refer to the *Methods* section), which makes the identified barriers and facilitators relevant. One reason for the relatively low numbers of digital mental health treatments provided during the COVID-19 pandemic could be the Swedish government’s decision to not use lockdown measures, meaning that health care was still provided face to face [47,48]. However, we do not present any data about digital mental health provision in other comparable countries, such as Denmark and Norway, which have similar public health systems but adopted different COVID-19 responses [48]. Thus, how and whether COVID-19 influenced digital mental health provision in these countries is an empirical question for further studies.

Fourth, the 2 open-ended questions were posed at the end of the survey, after asking 37 questions to be answered on a Likert scale, which could risk priming the responses. However, we deem this risk to be low because the abundance of Likert-scale questions would rather provide information overload than clear advice about possible barriers and facilitators for a respondent who has not considered them beforehand.

### Conclusions

Globally, the COVID-19 pandemic resulted in rapid deployment of various digital health solutions such as telehealth and videoconferencing to provide continued care despite distancing requirements. However, given the complex nature of digital mental health solution implementation, it is not probable that implementation based on sudden external pressure will be maintained. So far, few studies have examined the barriers to and facilitators of the implementation of digital mental health, and even fewer studies have examined the perspectives of decision makers. In this study, we report about various barriers and facilitators related to guidelines; health professionals; patients; incentives and resources; capacity for organization change; and social, political, and legal factors. Commonly reported barriers, by both implementers and nonimplementers, related to incentives and resources, whereas common facilitators were related to capacity for organizational change, and most differences were identified in relation to guidelines. Understanding similarities and differences can provide advice to future implementers of digital health regarding barriers and facilitators to take into consideration and inform the development of implementation strategies.

## Appendix

Multimedia Appendix 1 Started digital mental health treatments during 2018 and 2022. The file illustrates the number of started digital mental health treatments during 2018 and 2022. Data in the file are based on these studies [30,31,33].

Multimedia Appendix 2 Outcomes of summative content analysis and deductive analysis based on the Tailored Implementation in Chronic Diseases checklist. The file describes in detail the outcomes of the 2 coding procedures, listing the words and phrases, barriers, facilitators, and domains.

## Abbreviations

CBT: cognitive behavioral therapy
ICBT: internet-administered cognitive behavioral therapy
TICD: Tailored Implementation in Chronic Diseases

## References

[1] (2017). Depression and other common mental disorders: global health estimates. World Health Organization.

[2] Anwar N, Kuppili PP, Balhara YP (2017). Depression and physical noncommunicable diseases: the need for an integrated approach. WHO South East Asia J Public Health.

[3] Kessler RC, Demler O, Frank RG, Olfson M, Pincus HA, Walters EE, Wang P, Wells KB, Zaslavsky AM (2005). Prevalence and treatment of mental disorders, 1990 to 2003. N Engl J Med.

[4] Carlbring P, Andersson G, Cuijpers P, Riper H, Hedman-Lagerlöf E (2018). Internet-based vs. face-to-face cognitive behavior therapy for psychiatric and somatic disorders: an updated systematic review and meta-analysis. Cogn Behav Ther.

[5] Naslund JA, Aschbrenner KA, Araya R, Marsch LA, Unützer J, Patel V, Bartels SJ (2017). Digital technology for treating and preventing mental disorders in low-income and middle-income countries: a narrative review of the literature. Lancet Psychiatry.

[6] Lal S (2019). E-mental health: promising advancements in policy, research, and practice. Healthc Manage Forum.

[7] Riper H, Andersson G, Christensen H, Cuijpers P, Lange A, Eysenbach G (2010). Theme issue on e-mental health: a growing field in internet research. J Med Internet Res.

[8] Poletti B, Tagini S, Brugnera A, Parolin L, Pievani L, Ferrucci R, Compare A, Silani V (2021). Telepsychotherapy: a leaflet for psychotherapists in the age of COVID-19. A review of the evidence. Couns Psychol Q.

[9] Matsumoto K, Hamatani S, Nagai K, Sutoh C, Nakagawa A, Shimizu E (2020). Long-term effectiveness and cost-effectiveness of videoconference-delivered cognitive behavioral therapy for obsessive-compulsive disorder, panic disorder, and social anxiety disorder in Japan: one-year follow-up of a single-arm trial. JMIR Ment Health.

[10] Cuijpers P, Marks IM, van Straten A, Cavanagh K, Gega L, Andersson G (2009). Computer-aided psychotherapy for anxiety disorders: a meta-analytic review. Cogn Behav Ther.

[11] Donker T, van Straten A, Riper H, Marks I, Andersson G, Cuijpers P (2009). Implementation of internet-based preventive interventions for depression and anxiety: role of support? The design of a randomized controlled trial. Trials.

[12] Andersson G, Cuijpers P (2009). Internet-based and other computerized psychological treatments for adult depression: a meta-analysis. Cogn Behav Ther.

[13] Ellis LA, Augustsson H, Grødahl AI, Pomare C, Churruca K, Long JC, Ludlow K, Zurynski YA, Braithwaite J (2020). Implementation of e-mental health for depression and anxiety: a critical scoping review. J Community Psychol.

[14] Meurk C, Leung J, Hall W, Head BW, Whiteford H (2016). Establishing and governing e-mental health care in Australia: a systematic review of challenges and a call for policy-focussed research. J Med Internet Res.

[15] Apolinário-Hagen J, Kemper J, Stürmer C (2017). Public acceptability of e-mental health treatment services for psychological problems: a scoping review. JMIR Ment Health.

[16] Davies F, Shepherd HL, Beatty L, Clark B, Butow P, Shaw J (2020). Implementing web-based therapy in routine mental health care: systematic review of health professionals' perspectives. J Med Internet Res.

[17] Ganapathy A, Clough BA, Casey LM (2021). Organizational and policy barriers to the use of digital mental health by mental health professionals. Telemed J E Health.

[18] Schueller SM, Torous J (2020). Scaling evidence-based treatments through digital mental health. Am Psychol.

[19] Mendes-Santos C, Nunes F, Weiderpass E, Santana R, Andersson G (2022). Understanding mental health professionals' perspectives and practices regarding the implementation of digital mental health: qualitative study. JMIR Form Res.

[20] Ellis LA, Meulenbroeks I, Churruca K, Pomare C, Hatem S, Harrison R, Zurynski Y, Braithwaite J (2021). The application of e-mental health in response to COVID-19: scoping review and bibliometric analysis. JMIR Ment Health.

[21] Gagnon MP, Desmartis M, Labrecque M, Car J, Pagliari C, Pluye P, Frémont P, Gagnon J, Tremblay N, Légaré F (2012). Systematic review of factors influencing the adoption of information and communication technologies by healthcare professionals. J Med Syst.

[22] Helleman J, Kruitwagen ET, van den Berg LH, Visser-Meily JM, Beelen A (2020). The current use of telehealth in ALS care and the barriers to and facilitators of implementation: a systematic review. Amyotroph Lateral Scler Frontotemporal Degener.

[23] Kruse C, Fohn J, Wilson N, Nunez Patlan E, Zipp S, Mileski M (2020). Utilization barriers and medical outcomes commensurate with the use of telehealth among older adults: systematic review. JMIR Med Inform.

[24] Mileski M, Kruse CS, Catalani J, Haderer T (2017). Adopting telemedicine for the self-management of hypertension: systematic review. JMIR Med Inform.

[25] Neher M, Nygårdh A, Broström A, Lundgren J, Johansson P (2022). Perspectives of policy makers and service users concerning the implementation of eHealth in Sweden: interview study. J Med Internet Res.

[26] Williams MG, Stott R, Bromwich N, Oblak SK, Espie CA, Rose JB (2020). Determinants of and barriers to adoption of digital therapeutics for mental health at scale in the NHS. BMJ Innov.

[27] Brantnell A, Woodford J, Baraldi E, van Achterberg T, von Essen L (2020). Views of implementers and nonimplementers of internet-administered cognitive behavioral therapy for depression and anxiety: survey of primary care decision makers in Sweden. J Med Internet Res.

[28] Andersson G, Cuijpers P, Carlbring P, Riper H, Hedman E (2014). Guided internet-based vs. face-to-face cognitive behavior therapy for psychiatric and somatic disorders: a systematic review and meta-analysis. World Psychiatry.

[29] (2020). Nationella riktlinjer för vård vid depression och ångestsyndrom. Socialstyrelsen.

[30] (2020). Årsrapport: Svenska internetbehandlingsregistret, SibeR. Stockholm.

[31] (2021). Årsrapport Svenska internetbehandlingsregistret, SibeR. Stockholm.

[32] (2022). Behandlingsstarter per kvartal under 2022 (Q1-Q4). SibeR.

[33] Sundquist J, Ohlsson H, Sundquist K, Kendler KS (2017). Common adult psychiatric disorders in Swedish primary care where most mental health patients are treated. BMC Psychiatry.

[34] Hsieh HF, Shannon SE (2005). Three approaches to qualitative content analysis. Qual Health Res.

[35] Elo S, Kyngäs H (2008). The qualitative content analysis process. J Adv Nurs.

[36] Flottorp SA, Oxman AD, Krause J, Musila NR, Wensing M, Godycki-Cwirko M, Baker R, Eccles MP (2013). A checklist for identifying determinants of practice: a systematic review and synthesis of frameworks and taxonomies of factors that prevent or enable improvements in healthcare professional practice. Implement Sci.

[37] Synonymer.se. Sinovum Media.

[38] Elo S, Kaariainen M, Kanste O, Polkki T, Utriainen K, Kyngas H (2014). Qualitative content analysis: a focus on trustworthiness. SAGE Open.

[39] Oliver K, Innvar S, Lorenc T, Woodman J, Thomas J (2014). A systematic review of barriers to and facilitators of the use of evidence by policymakers. BMC Health Serv Res.

[40] Bach-Mortensen AM, Lange BC, Montgomery P (2018). Barriers and facilitators to implementing evidence-based interventions among third sector organisations: a systematic review. Implement Sci.

[41] Orton L, Lloyd-Williams F, Taylor-Robinson D, O'Flaherty M, Capewell S (2011). The use of research evidence in public health decision making processes: systematic review. PLoS One.

[42] Kok G, Gottlieb NH, Peters GJ, Mullen PD, Parcel GS, Ruiter RA, Fernández ME, Markham C, Bartholomew LK (2016). A taxonomy of behaviour change methods: an intervention mapping approach. Health Psychol Rev.

[43] Grimshaw JM, Eccles MP, Lavis JN, Hill SJ, Squires JE (2012). Knowledge translation of research findings. Implement Sci.

[44] Jacob C, Sanchez-Vazquez A, Ivory C (2020). Social, organizational, and technological factors impacting clinicians' adoption of mobile health tools: systematic literature review. JMIR Mhealth Uhealth.

[45] (2022). Mental health tech report Sweden 2022. Skåne Startups.

[46] Wong B, Buttigieg S, Brito DV (2021). Preparing the public health workforce for digital health futures: the case for digital health training and capacity building. Eur J Public Health.

[47] Ludvigsson JF (2023). How Sweden approached the COVID-19 pandemic: summary and commentary on the national commission inquiry. Acta Paediatr.

[48] Laage-Thomsen J, Frandsen SL (2022). Pandemic preparedness systems and diverging COVID-19 responses within similar public health regimes: a comparative study of expert perceptions of pandemic response in Denmark, Norway, and Sweden. Global Health.

