# Supplementary figures and images for: Barriers to and Facilitators of the Implementation of Digital Mental Health Interventions as Perceived by Primary Care Decision Makers: Content Analysis of Structured Open-Ended Survey Data
Source: JMIR Hum Factors. 2023 Jun 26;10:e44688. doi: 10.2196/44688 (PMC10337378; doi:10.2196/44688)

**Multimedia Appendix 1. Started digital mental health treatments during 2018 and 2022**


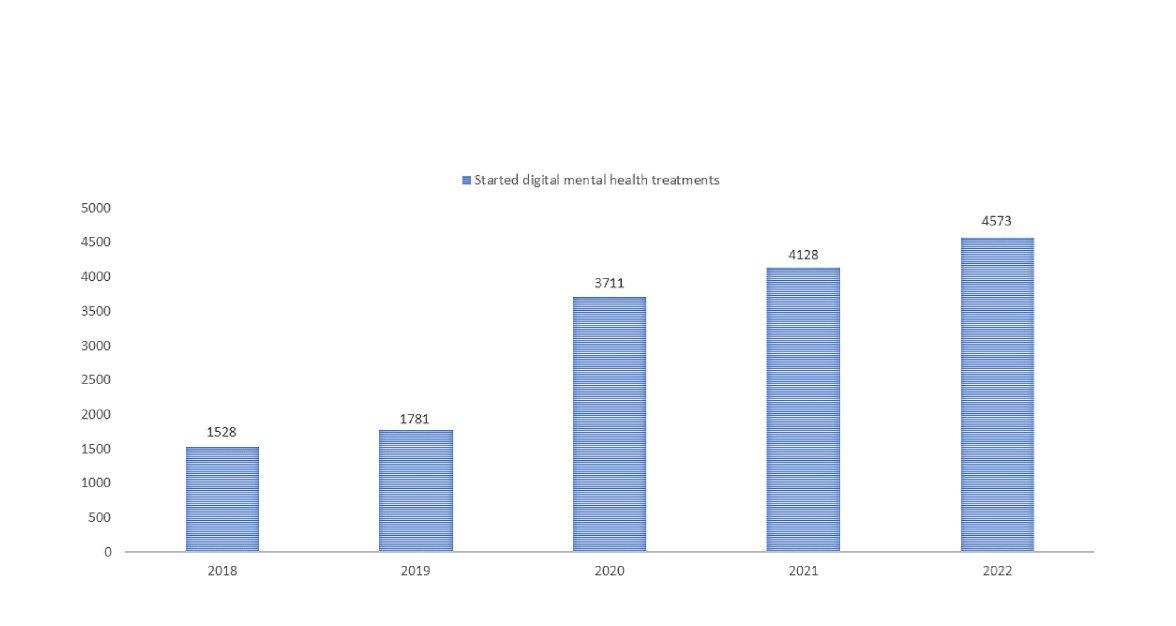

Supplement: Multimedia Appendix 1 [file humanfactors_v10i1e44688_app1.docx]
