# Supplementary material for: Barriers to and Facilitators of the Implementation of Digital Mental Health Interventions as Perceived by Primary Care Decision Makers: Content Analysis of Structured Open-Ended Survey Data
Source: JMIR Hum Factors. 2023 Jun 26;10:e44688. doi: 10.2196/44688 (PMC10337378; doi:10.2196/44688)
Supplement: Multimedia Appendix 2 [file humanfactors_v10i1e44688_app2.docx]

| **Appendix 2. Codebook showing the** **outcomes of summative content analysis and deductive analysis based on the TICD checklist** | | |
| --- | --- | --- |
| Summative content analysis | Deductive analysis | |
| **Words/phrases mentioned by respondents (B=barrier, F=facilitator)** | **Barriers & facilitators** | **Domains** |
| Lack of information about effect (B) | Quality of evidence  supporting the recommendation (How confident we are in the estimates of effects) | Guidelines |
| Same outcomes as with traditional treatment (F) | Strength of recommendation (How confident we are that the desirable effects of adherence to the recommendation outweigh the undesirable effects) |  |
| Can help patients (F) |  |  |
| Suitable for patients that do not need face to face treatment (F) |  |  |
| ICBT not suitable for all patients (B) |  |  |
| Patient safety unclear (B) |  |  |
| Added value unclear (B) |  |  |
| Not clear what ICBT should be used for (B) | Clarity (The clearness of the target population, the settings in which the recommendation is to be used and the recommended action) |  |
| Inhumane treatment (B) | Cultural  appropriateness (The extent to which the recommendation is suitable in the social context where it is being implemented) |  |
| Access to ICBT (F) | Accessibility of the intervention (The extent to which the recommended clinical intervention is accessible) |  |
| Lack of access to ICBT (B) |  |  |
| Increases patients’ access to treatment (F) |  |  |
| Patient can decide over bookings (F) | Feasibility (The extent to which the recommended clinical intervention is practical) |  |
| Technical problems (B) |  |  |
| Low flexibility (B) |  |  |
| Easy to use (F) |  |  |
| Saves time for patients (F) |  |  |
| Difficult to identify symptoms via ICBT (B) |  |  |
| Requires work with technology (B) | Compatibility (The extent to which the recommended behavior fits with current practice) |  |
| Home care (F) |  |  |

| **Appendix 2. Continued** | | |
| --- | --- | --- |
| Summative content analysis | Deductive analysis | |
| **Words/phrases mentioned by respondents (B=barrier, F=facilitator)** | **Barriers & facilitators** | **Domains** |
| Difficult to identify suitable patients (B) | Effort (The amount of effort required to change or adhere) | Guidelines |
| Not possible to trial (B) | Trialability (The ability to try out the recommended behavior) |  |
| Creates room for other patients (F) | Observability (The degree to which benefits of the recommended behavior are visible) |  |
| Lack of knowledge by health professionals (B) | Awareness and  familiarity with the recommendation (The extent to which the targeted  healthcare professionals are  aware of and familiar with the recommendation) | Health professionals |
| Requires training of health professionals (B) | Skills needed to adhere (The extent to which the targeted  health professionals have skills that they need to adhere) |  |
| Health professionals lack experience in ICBT (B) |  |  |
| Health professionals’ negative attitudes towards ICBT (B) | Agreement with the recommendation (The extent to which the targeted  healthcare professionals agree with the recommendation) |  |
| Health professionals not interested (B) | Intention and  motivation (The extent to which the targeted  healthcare professionals intend to adhere and are motivated to do so) |  |
| Health professionals interested (F) |  |  |
| Health professionals motivated (F) |  |  |
| Health professionals not motivated (B) |  |  |
| Health professionals prefer to meet the patient face-to-face (B) |  |  |

| **Appendix 2. Continued** | | |
| --- | --- | --- |
| Summative content analysis | Deductive analysis | |
| **Words/phrases mentioned by respondents (B=barrier, F=facilitator)** | **Barriers & facilitators** | **Domains** |
| Requires new and changed work routines (B) | Nature of the behavior (Characteristics of the behavior, e.g., frequency of  performance for a patient,  frequency of performance for a  population of patients, the degree  of habit or automaticity) | Health professionals |
| Requires routines to work with computer (B) |  |  |
| Knowledge among the general public about ICBT (F) | Patient beliefs and know-ledge (Patients’ beliefs or knowledge or ability to learn) | Patients |
| Information to patients (F) |  |  |
| Lack of information to patients (B) |  |  |
| Patients lack of knowledge (B) |  |  |
| Patients prefer face-to-face treatment (B) | Patient preferences (Patient’s values in relationship to professional values) |  |
| Patients not interested (B) | Patient motivation (The targeted healthcare professionals’ ability or perceived ability to motivate patients to adhere and relation with actual patient motivation) |  |
| Patients interested (F) |  |  |
| Patients lack routines to use computers (B) | Patient behavior (Patient behaviors that motivate or demotivate adherence) |  |
| Patients have routines to use computers (F) |  |  |
| Lack of resources (B) | Availability of necessary  Resources (The extent to which the resources that are needed to adhere are available) | Incentives and resources |
| Available resources (F) |  |  |
| Lack of trained health professionals (B) |  |  |
| Lack of health professionals (B) |  |  |
| Licensing costs (B) |  |  |

| **Appendix 2. Continued** | | |
| --- | --- | --- |
| Summative content analysis | Deductive analysis | |
| **Words/phrases mentioned by respondents (B=barrier, F=facilitator)** | **Barriers & facilitators** | **Domains** |
| Issues with internet connection (B) | Availability of supporting infrastructure (The extent to which the infrastructure that is needed to adhere is available) | Incentives and resources |
| Access to technology that works (F) |  |  |
| No access to computers (B) |  |  |
| Patients lack access to technology (B) |  |  |
| Computers that patients can use here (F) |  |  |
| Reimbursement system does not support ICBT (B) | Financial incentives and  disincentives (The extent to which patients, individual health professionals and organisations have financial  incentives or disincentives to  adhere) |  |
| Reimbursement system that supports ICBT (F) |  |  |
| Resources can be saved (F) |  |  |
| ICBT does not entail enough time saving for psychologists (B) |  |  |
| ICBT entails a cost (B) |  |  |
| Inexpensive solution (F) |  |  |
| Cost-effectiveness (F) |  |  |
| Entails low cost (F) |  |  |
| Less time required from the therapist (F) | Non-financial incentives and disincentives (The extent to which patients, individual health professionals  and organisations have  nonfinancial incentives or  disincentives to adhere) |  |
| Que to CBT treatment (F) |  |  |
| Good spread of information to staff (F) | Information system (The extent to which the information system facilitates or hinders adherence) |  |
| Poor information to health professionals (B) |  |  |
| Information to leadership (F) |  |  |
| Improved IT system in the organization (F) |  |  |
| Information to health professionals (F) |  |  |
| IT systems are incompatible (B) |  |  |
| The surrounding technology does not work (B) |  |  |

| **Appendix 2. Continued** | | |
| --- | --- | --- |
| Summative content analysis | Deductive analysis | |
| **Words/phrases mentioned by respondents (B=barrier, F=facilitator)** | **Barriers & facilitators** | **Domains** |
| As a private actor we should be able to offer the treatment by ourselves but it is difficult (B) | Mandate, authority,  accountability (The mandate, authority and  accountability for making  necessary changes) | Capacity for organizational change |
| Central decision to use ICBT (F) |  |  |
| Lack of routines to use computers (B) | Organizational readiness (The extent to which the organization is ready to adopt the intervention) |  |
| Difficult to start using new treatments (B) |  |  |
| Existing tradition (B) |  |  |
| Lack of routines to implement ICBT (B) |  |  |
| Leadership not interested (B) | Capable leadership (The extent to which clinical leaders or managers are capable of making necessary changes) |  |
| Leadership interested (F) |  |  |
| Leadership lacks knowledge (B) |  |  |
| Leadership has knowledge (F) |  |  |
| Leadership lacks information (B) |  |  |
| Leadership has information (F) |  |  |
| Leadership is scared (B) |  |  |
| Support in the organization (F) | Relative strength of  supporters and  opponents (The extent of support and opposition to necessary changes) |  |
| No strategy for treatment with mental health issues (B) | Regulations, rules,  Policies (The extent to which  organisational regulations, rules or policies facilitate or hinder necessary changes) |  |
| Education for leadership (F) | Assistance for  organisational changes (The extent to which external  support is needed and available for necessary changes) |  |
| Lack of education for leadership (B) |  |  |
| Training for health professionals (F) |  |  |
| Lack of training for health professionals (B) |  |  |
| Inadequate support with implementation (B) |  |  |
| Support with implementation (F) |  |  |
| Lack of technical support (B) |  |  |

| **Appendix 2. Continued** | | |
| --- | --- | --- |
| Summative content analysis | Deductive analysis | |
| **Words/phrases mentioned by respondents (B=barrier, F=facilitator)** | **Barriers & facilitators** | **Domains** |
| Bureaucracy connected to healthcare systems (B) | The healthcare system (The extent to which the healthcare system hinders or facilitates adherence to the intervention) | Social, political and legal factors |
| Healthcare organization (B) |  |  |
| All healthcare units cannot handle ICBT (B) |  |  |
| The medical records system (B) |  |  |
| General maturity for technical devices in healthcare (F) |  |  |
| Care contract does not allow ICBT (B) | Contracts (The extent to which contracts may affect implementation of  necessary changes) |  |
